# Uremic Toxin-Induced Pathological Signaling in Sinus Node Cells Is Attenuated by Probenecid

**DOI:** 10.1159/crm/abtag008

**Published:** 2026-09-07

**Authors:** Dal-Ah Kim, Chor Ho Jo, Mi-Ran Lee, Jung-Hwa Ryu, Kyoung Hye Kong

**Affiliations:** a Medical Research Center, Ewha Womans University College of Medicine, Seoul, Republic of Korea; b Department of Biomedical Laboratory Science, Jungwon University, Chungbuk, Republic of Korea; c Department of Internal Medicine, Ewha Womans University College of Medicine, Seoul, Republic of Korea; d Inflammation-Cancer Microenvironment Research Center, Ewha Womans University College of Medicine, Seoul, Republic of Korea

**Keywords:** Chronic kidney disease, Sinus nodal cell, Uremic toxins, Pacemaker cell signaling

## Abstract

**Introduction:**

Uremic toxins, including indoxyl sulfate (IS) and p-cresyl sulfate (PCS), are well-recognized contributors to cardiac fibrosis and maladaptive remodeling in chronic kidney disease (CKD). Although their roles in ventricular and vascular pathology have been extensively studied, their effects on sinus node cells and the associated molecular alterations remain poorly defined. Understanding how the uremic milieu affects sinus node cell homeostasis may provide insight into CKD-associated conduction abnormalities.

**Methods:**

To investigate uremic toxin-induced stress responses, H9C2 cardiomyocytes were used as a comparative cardiac stress-responsive model, whereas primary sinus node cells served as the principal pacemaker-relevant model. Both cell types were treated with IS and PCS to compare shared cardiac stress signaling with sinus node cell-associated remodeling responses. Profibrotic, pro-apoptotic, and stress-related signaling pathways were assessed using protein and mRNA analyses. Activation of stress kinases, including p38 and ERK, was evaluated to define downstream signaling mechanisms. In parallel, a CKD mouse model was generated by 2-week adenine feeding, and sinus node cells were isolated for molecular analysis. The protective effects of probenecid were examined in cultured cells and ex vivo using primary sinus node cells isolated from adenine-fed mice, with particular attention to remodeling-associated molecular markers and pacemaker identity marker expression.

**Results:**

IS and PCS induced profibrotic, pro-apoptotic, and remodeling-associated signaling in both H9C2 cardiomyocytes and primary sinus node cells, indicating activation of shared cardiac stress pathways. Conclusions regarding pacemaker-relevant molecular alterations were based primarily on findings obtained from primary sinus node cells, including cells isolated from 2-week adenine-fed mice. In primary sinus node cells, uremic toxin exposure increased fibronectin accumulation, altered the Bax/Bcl-2 balance, and activated p38 and ERK signaling. TBX3 expression was preserved, suggesting that these molecular changes occurred without overt loss of this pacemaker identity marker. These findings indicate activation of apoptosis-associated, profibrotic, and stress-responsive molecular alterations rather than direct evidence of functional pacemaker impairment. Consistent with in vitro findings, sinus node cells isolated from 2-week adenine-fed mice exhibited increased expression of remodeling-associated molecular markers. Probenecid treatment attenuated toxin-associated fibronectin accumulation, Bax/Bcl-2 imbalance, and stress kinase activation in primary sinus node cells. Similar attenuation of these remodeling-associated molecular marker changes was observed in sinus node cells isolated from adenine-fed mice following ex vivo treatment with probenecid.

**Conclusion:**

These findings suggest that uremic toxin-driven stress signaling is associated with molecular remodeling signatures in sinus node cells under CKD-related conditions. However, the functional consequences of these molecular alterations were not assessed. Probenecid attenuated uremic toxin-associated profibrotic, pro-apoptotic, and stress kinase signaling, suggesting that targeting uremic toxin-driven molecular stress pathways may represent a potential strategy to preserve sinus node cell homeostasis in CKD.

## Introduction

Chronic kidney disease (CKD) is strongly linked to elevated cardiovascular morbidity and mortality [1], and accumulating evidence shows that the uremic milieu directly drives structural and electrical changes in the heart [2]. Among the toxins retained in CKD, the protein-bound solutes indoxyl sulfate (IS) and p-cresyl sulfate (PCS) are recognized as key pathogenic mediators [3]. These uremic toxins induce oxidative stress, mitochondrial dysfunction, and fibro-inflammatory signaling in cardiac and vascular tissues, thereby accelerating maladaptive remodeling processes [4, 5]. Mechanistically, IS activates TGF-β, MAPK, and NF-κB pathways, promoting extracellular matrix accumulation, apoptosis and impaired cardiomyocyte survival [6], while PCS similarly contributes to cardiac fibrosis and hypertrophy through ROS-dependent stress kinase activation [7].

Despite extensive characterization of uremic toxin-induced ventricular toxicity [8], their impact on the cardiac conduction system, particularly the sinoatrial node (SAN), remains poorly defined. Patients with CKD have a substantially higher incidence of bradyarrhythmias, conduction abnormalities, and permanent pacemaker implantation than the general population, and progressive renal dysfunction has been associated with worsening cardiac conduction abnormalities, suggesting that CKD-related systemic factors may adversely affect sinus node function [9]. The SAN is a metabolically demanding structure that is highly vulnerable to metabolic and oxidative disturbances, making it particularly susceptible to the uremic milieu. However, mechanistic studies addressing whether IS or PCS alter pacemaker cell identity or induce profibrotic molecular changes in sinus node cells remain scarce.

Probenecid, originally characterized as an organic anion transporter inhibitor, has recently gained attention as a cardiomodulatory compound [10]. It enhances intracellular Ca^2+^ handling through activation of transient receptor potential vanilloid 2 (TRPV2) channels and confers protection against stress-induced cellular injury in cardiomyocytes [11]. In addition, probenecid has been reported to attenuate pathological cardiac remodeling and stabilize electrophysiological activity under disease conditions [6, 10]. Nonetheless, its potential to attenuate uremic toxin-associated molecular stress responses in pacemaker cells has not been investigated.

In this study, we examined how IS and PCS regulate profibrotic, apoptotic, and stress-related signaling in two complementary cellular systems. H9C2 cardiomyocytes were included as a well-established comparative model of cardiac stress-responsive signaling, not as a surrogate for pacemaker cells or sinus node automaticity. Primary sinus node cells were used as the principal disease-relevant model to evaluate pacemaker-relevant molecular alterations. This design allowed us to distinguish generalized cardiac stress responses from pacemaker-relevant molecular alterations in primary sinus node cells. We further examined whether probenecid attenuates toxin-induced molecular stress responses in these systems. To provide ex vivo support for these findings, sinus node cells were additionally isolated from 2-week adenine-fed mice and compared with cells from healthy controls. Through combined protein and mRNA analyses, we sought to determine whether uremic toxin-associated molecular alterations are also present in pacemaker cells and to identify the signaling pathways through which probenecid modulates these responses, thereby providing insight into uremia-associated molecular alterations in sinus node cells and potential mechanisms relevant to pacemaker cell homeostasis in renal disease.

## Methods

### Experimental Design and Treatment Groups

To simulate the uremic milieu associated with CKD, IS (Sigma Chemical Co., St. Louis, MO) or PCS (Apexbio Technology LLC, Houston, TX, USA) were used as representative protein-bound uremic toxins. Probenecid (Invitrogen, Thermo Scientific Pierce, Rockford, IL, USA) was administered as a pharmacological protective agent. For in vitro experiments, both H9C2 cardiomyocytes and primary sinus node cells isolated from normal mouse hearts were assigned to three experimental groups: (i) untreated control, (ii) IS (50 μg/mL for H9C2, 5 μg/mL for sinus node cells) or PCS (100 μm), and (iii) IS or PCS co-treated with probenecid (1 mm). Different IS concentrations were used for H9C2 cardiomyocytes and primary sinus node cells to account for cell type-specific tolerance. H9C2 cardiomyocytes were treated with 50 μg/mL IS based on their higher tolerance and previous H9C2-based studies using comparable or higher IS concentrations to evaluate uremic toxin-induced cardiac stress signaling. In contrast, primary sinus node cells showed greater vulnerability to IS exposure during preliminary culture observations; therefore, 5 μg/mL IS was selected as a working concentration that allowed assessment of toxin-associated molecular responses while minimizing nonspecific cell loss. H9C2 cardiomyocytes were used to evaluate generalized cardiac stress signaling, whereas primary sinus node cells were used to assess pacemaker-relevant molecular alterations. Therefore, conclusions regarding molecular alterations relevant to sinus node biology were drawn primarily from primary sinus node cell experiments, including cells isolated from 2-week adenine-fed mice. All treatments were performed for 48 h under identical culture conditions, after which cells were harvested for subsequent analyses according to the experimental endpoints.

For ex vivo experiments, primary sinus node cells were also isolated from the hearts of mice fed a 0.2% adenine diet for 2 weeks to generate an early uremic stress model. Cells were divided into three experimental groups: (i) normal sinus node cells, (ii) sinus node cells isolated from 2-week adenine-fed mice, and (iii) sinus node cells isolated from 2-week adenine-fed mice and treated with probenecid (1 mm) for comparative analysis of uremia-associated molecular alterations.

### Isolation and Culture of Sinus Nodal Cells

All animal procedures were approved by the Institutional Animal Care and Use Committee of Ewha Womans University (No. 21-020) and conducted in accordance with the National Institutes of Health (NIH) Guide for the Care and Use of Laboratory Animals. Adult male C57BL/6 mice (≥8 weeks old; Orient Bio, Seongnam, Korea) were used for all experiments. Briefly, mice were anesthetized via intraperitoneal injection of a general anesthetic, after which the thoracic cavity was opened and the heart was rapidly excised. The right atrial tissue, including the SAN region, was dissected into small fragments and digested at 37°C for 30 min in Dulbecco’s phosphate-buffered saline (PBS) containing collagenase type II (2 mg/mL; Worthington Biochemical Corp., Lakewood, NJ, USA) with gentle agitation. Following enzymatic digestion, the cell suspension was centrifuged at 5,000 *g* for 5 min, washed twice with collagenase-containing Dulbecco’s PBS, and finally resuspended in culture medium consisting of 20% fetal bovine serum (FBS) and 1% penicillin-streptomycin. The cells were plated on gelatin-coated culture dishes and maintained at 37°C in a humidified atmosphere of 5% CO_2_. The culture medium was replaced every 2 days, and the cells typically reached ∼80% confluence within 7 days [12].

At this time, IS was added to the culture medium for treatment groups, whereas control cells received vehicle alone. Unless otherwise specified, cells were harvested after 48 h of treatment when cultures reached near confluence (∼100%). The use of a high fetal bovine serum concentration (20%) was found to be well tolerated by sinus nodal cells, promoting pacemaker cell growth without fibroblast contamination.

### Immunofluorescence

When sinus node cells reached approximately 80% confluence on coverslips, they were rinsed twice with PBS and fixed with 10% formalin in PBS (pH 7.4) for 30 min at room temperature. To block nonspecific antibody binding, cells were incubated with blocking buffer containing 5% bovine serum albumin and 0.1% Triton X-100 for 30 min at room temperature. After blocking, cells were incubated overnight at 4°C with the primary antibody against TBX3 (rabbit polyclonal, 1:200 dilution; Abcam, Cambridge, UK) diluted in blocking buffer. The following day, cells were washed three times with PBS and incubated for 1 h at room temperature with a fluorescent secondary antibody (goat anti-rabbit IgG conjugated with Alexa Fluor^®^ 594; Invitrogen, Thermo Fisher Scientific, Waltham, MA, USA). After final PBS washes, nuclei were counterstained with DAPI (1 µg/mL) for 5 min, and coverslips were mounted using Gel Mount Aqueous mounting medium (Sigma-Aldrich, St. Louis, MO, USA). Fluorescent images were captured using a Nikon D1X digital fluorescence microscope (Carl Zeiss, Oberkochen, Germany) under identical exposure settings for all groups.

### Flow Cytometry

Flow cytometric analysis was performed to quantify the proportion of TBX3-positive cells, a specific marker for sinus node identity. Sinus node cells were harvested using 0.25% trypsin-EDTA (Gibco, Grand Island, NY, USA) and washed twice with cold PBS containing 0.1% bovine serum albumin at 4°C. Cells were incubated with a primary anti-TBX3 antibody (rabbit polyclonal, 1:500; Abcam, Cambridge, UK) for 30 min at room temperature in the dark, followed by two washes with PBS. Subsequently, cells were incubated with a FITC-conjugated goat anti-rabbit IgG secondary antibody (1:1,000; Invitrogen, Thermo Fisher Scientific, Waltham, MA, USA) for 15 min at room temperature in the dark. After two final washes, the cell suspension was diluted in 400 μL of PBS, and samples were analyzed within 1 h using a NovaSampler Pro Combo (Agilent Technologies, Santa Clara, CA, USA) equipped with NovoExpress software. Data were acquired from at least 10,000 events per sample, and the percentage of TBX3-positive cells was determined by gating against isotype-matched controls.

### Western Blotting

The cultured cell pellets were lysed in radioimmunoprecipitation assay lysis buffer (1×) supplemented with EDTA (GenDEPOT, Katy, TX, USA) and a complete protease inhibitor cocktail (GenDEPOT, Katy, TX, USA) and quantified using Pierce BCA Protein Assay Kit (Thermo Fisher Scientific Inc., Rockford, IL, USA). Equal amounts of protein were separated by SDS-PAGE and then transferred to PVDF membranes (Bio-Rad Laboratories Inc., Hercules, CA, USA). Following protein transfer, the PVDF membranes were sectioned horizontally according to the expected molecular weights of the target proteins before blocking and primary antibody incubation to allow independent immunoblotting of each target protein. Each membrane strip wase incubated overnight at 4°C with the following primary antibodies: anti-TBX3 (TBX3; a sinus node marker; ab89220, Abcam), anti-fibronectin (rabbit, 1:1,000; Dako), anti-E-cadherin (mouse, 1:1,000; BD Biosciences), anti-α-SMA (rabbit, 1:1,000; Abcam), anti-p-p38 (rabbit, 1:500; Cell Signaling), anti- p38 (rabbit, 1:1,000; Cell Signaling), and anti-β-actin (mouse, 1:1,000; Sigma Chemical Co.). Protein bands were detected using an enhanced chemiluminescence reagent (Thermo Scientific Pierce, Rockford, IL, USA) and Luminescent Image Analyzer 3 (Bio-Rad Laboratories Inc., Hercules, CA, USA). After chemiluminescent detection, no additional cropping or digital manipulation of the blot images was performed. Band densities were quantified using Image J v.1.49 (NIH, Bethesda, MD, USA). The original uncropped Western blot images are provided as online supplementary Figures S1–S3 (for all online suppl. material, see https://doi.org/10.1159/crm/abtag008).

### Quantitative Reverse Transcriptase Polymerase Chain Reaction Analysis

Total mRNA was isolated from H9C2 cardiomyocytes and primary sinus node cells using RNeasy mini kit (Qiagen, Hilden, Germany) according to the manufacturer’s protocol. Complementary cDNA was synthesized using the Cycle Script™ RT PreMix (dN6) (Bioneer, Daejeon, Republic of Korea). Quantitative real-time PCR was performed on a QuantStudio3 real-time PCR (Applied Biosystems, Foster City, CA, USA) using amfiSure qGreen Q-PCR Master Mix (2×) with High ROX (GenDEPOT, Baker, TX, USA). Each experiment was independently repeated three times.

For mouse sinus node cells, the following primers were used: *fibronectin*: forward 5’-GAT GCA CCG ATT GTC AAC AG-3’, reverse 5’-ACT CTG ATC AGC ATG GAC CA-3’; *α-SMA*: forward 5’-ACT GGG ACG ACA TGG AAA AG-3’, reverse 5’-GCT GCT TCA GAC ACT TGC ACA A-3’; *ANP*: forward 5’-GAA CCT GCT AGA CCA CCT-3’, reverse 5’-CCT AGT CCA CTC TGG GCT-3’; *BNP*: forward 5’-AAG CTG CTG GAG CTG ATA AGA, reverse 5’-GTT ACA GCC CAA ACG ACT GAC-3’; *β-MHC*: forward 5’-CTC AAG CTG CTC AGC AAT CTA TTT-3’, reverse 5’-GGA GCG CAA GTT TGT CAT AAG T-3’; *HPRT*: forward 5’-CAG ACT GAA GAG CTA CTG TAA TG-3’, reverse 5’-CCA GTG TCA ATT ATA TCT TCA AC-3’.

For rat H9C2 cardiomyocytes, the following primers were used: *fibronectin*: forward 5’-CAT GGC TTT AGG CGA ACC A-3’, reverse 5’-CAT CTA CAT TCG GCA GGT ATG G-3’; *α-SMA*: forward 5’-GAC CCT GAA GTA TCC GAT AGA ACA G-3’, reverse 5’-CAC GCG AAG CTC GTT ATA GAA G-3’; *ANP*: forward 5’-ATG GGC TCC TTC TCC ATC AC-3’, reverse 5’-TTC ATC GGT ATG CTC GCT CA-3’; *BNP*: forward 5’-TGG GAA GTC CTA GCC AGT CT-3’, reverse 5’-GAT CCG GTC TAT CTT CTG CC-3’; *β-MHC*: forward 5’-CTA GGA GGC GGA GGA ACA G-3’, reverse 5’-CTT GGC GCC AAT GTC ACG-3’; *β-actin*: forward 5’-ACC ACA GTC CAT GCC ATC AC-3’, reverse 5’-TCC ACC ACC CTG TTG CTG TA-3’.

Relative mRNA expression level of fibronectin, α-SMA, ANP, BNP, and β-MHC were calculated after normalization to the internal control genes (HPRT for mouse, actin for rat) using the comparative 2^−ΔΔCT^ method. All data are presented as mean ± standard error of the mean (SEM) from at least three independent experiments.

### Statistical Analysis

All statistical analyses were performed using GraphPad Prism 8.0 (GraphPad Software, San Diego, CA, USA). Data are presented as the mean ± SEM. Comparisons among three or more groups were performed using one-way analysis of variance followed by Tukey’s multiple comparisons test. A *p* value <0.05 was considered statistically significant.

## Results

### Primary Sinus Node Cells Isolated from Normal Mouse Hearts Preserve Pacemaker Cell Identity

To confirm that the cells isolated from normal mouse hearts represent SAN-derived pacemaker cells, immunofluorescence staining was performed to examine the expression of TBX3, a transcription factor essential for SAN specification and maintenance. The cultured cells exhibited robust TBX3 immunoreactivity, with predominant nuclear and perinuclear localization overlapping with DAPI-stained nuclei, indicating enrichment of nodal-lineage cardiomyocytes (shown in Fig. 1a). The sustained expression pattern of TBX3 in cultured cells supports the preservation of pacemaker cell identity following isolation.

**Fig. 1.**
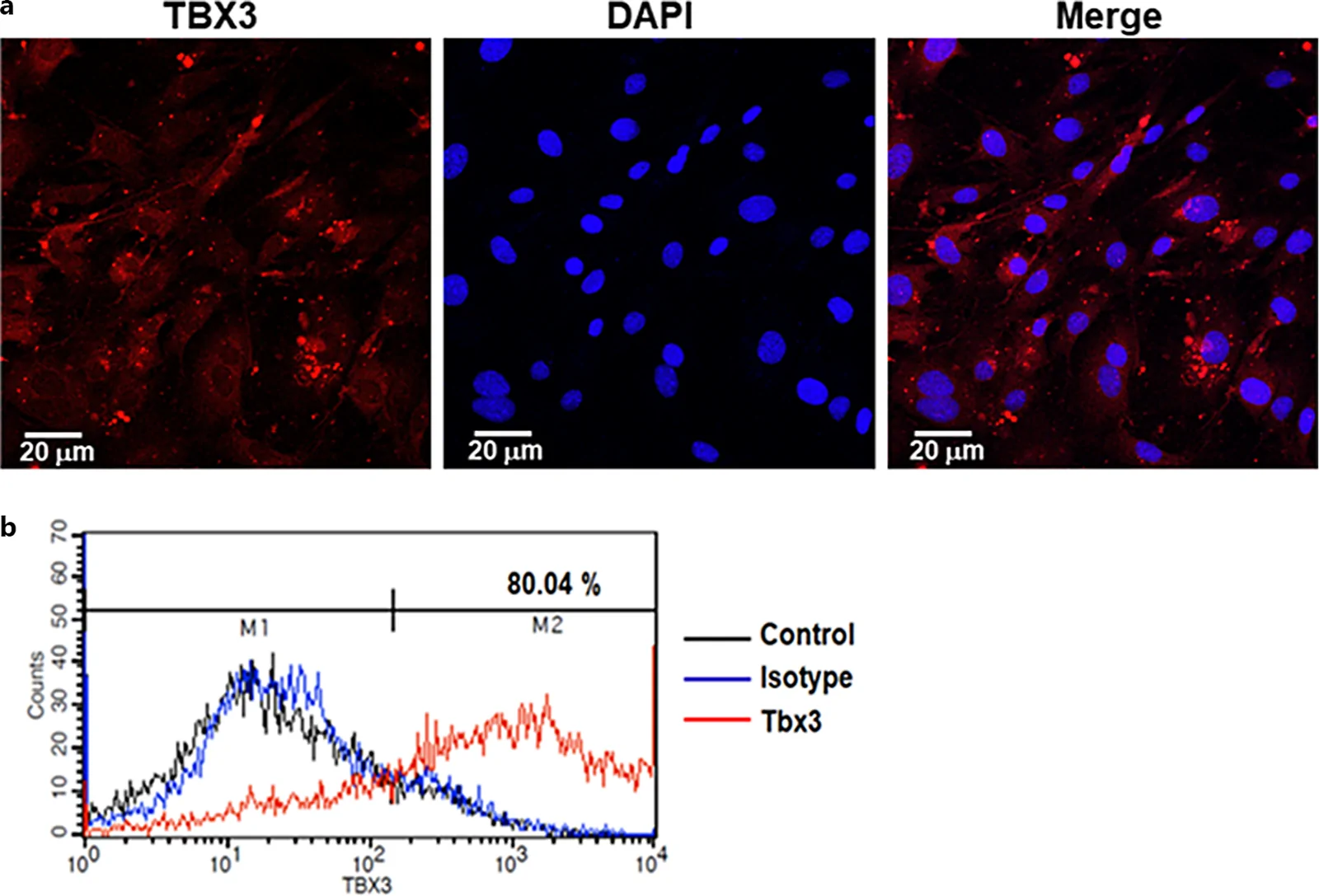
Characterization of primary sinus node cells isolated from normal mouse hearts. **a** Immunofluorescence staining of TBX3 in primary sinus node cells cultured from normal mouse cardiac tissue. TBX3 immunoreactivity is shown in red, and nuclei were counterstained with DAPI in blue. Scale bar: 20 μm. **b** Flow cytometric analysis of TBX3 expression in cultured sinus node cells. The TBX3-positive population is shown as a red histogram and was compared with isotype control and untreated control cells. The percentage of TBX3-positive cells is indicated in the M2 gate.

Flow cytometric analysis further quantitatively validated the nodal phenotype of the isolated cell population. A substantial proportion of cells (80.04%) were TBX3-positive, showing clear separation from both isotype controls and untreated negative controls (shown in Fig. 1b). Together, these results demonstrate that primary cells isolated from the murine SAN maintain characteristic pacemaker cell identity under basal culture conditions.

H9C2 cardiomyocytes provide comparative evidence of generalized cardiac stress signaling, whereas primary sinus node cells exhibit pacemaker-relevant molecular alterations after IS and PCS exposure. To distinguish generalized cardiomyocyte stress responses from molecular alterations relevant to sinus node biology, parallel experiments were performed using H9C2 cardiomyocytes and primary sinus node cells. H9C2 cardiomyocytes were used solely as a comparative model of generalized stress-responsive cardiac signaling, whereas primary sinus node cells served as the principal model for evaluating molecular changes relevant to pacemaker biology.

In H9C2 cardiomyocytes, IS (50 µg/mL) and PCS (100 µm) significantly increased fibronectin expression and Bax levels while reducing Bcl-2 expression. These changes were accompanied by decreased E-cadherin expression and increased p-p38/p38 and p-ERK/ERK ratios (shown in Fig. 2). Collectively, these findings indicate that IS and PCS activate generalized profibrotic, apoptosis-associated, and stress-responsive molecular signaling in H9C2 cardiomyocytes. Because H9C2 cells are not pacemaker cells, these results were interpreted solely as comparative evidence of generalized cardiomyocyte stress responses and were not used to support conclusions regarding functional impairment or molecular alterations specific to sinus node cells.

**Fig. 2.**
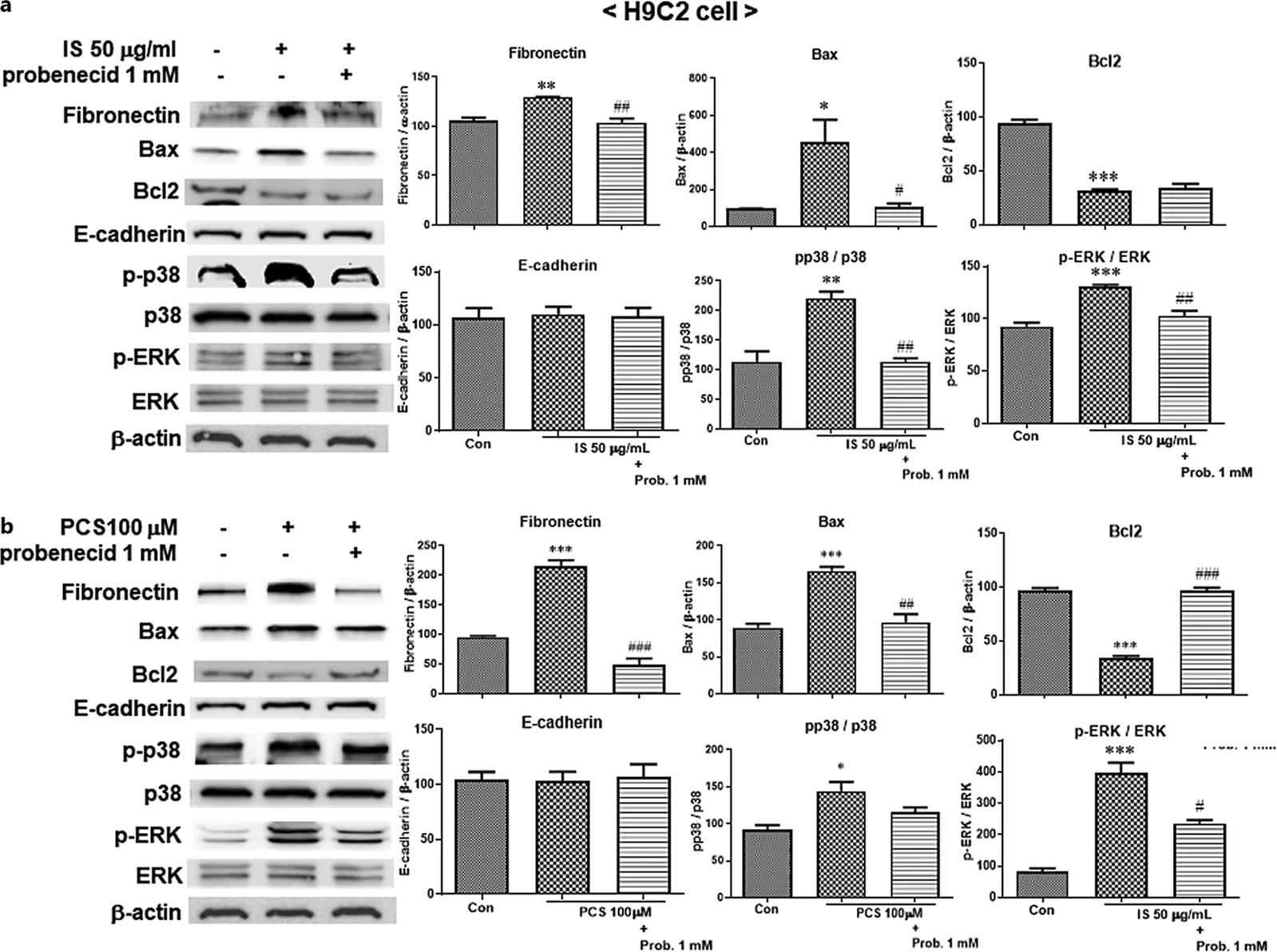
Effects of indoxyl sulfate, p-cresyl sulfate, and probenecid on profibrotic, apoptosis-associated, and stress-related molecular markers in H9C2 cardiomyocytes used as a comparative cardiac stress-responsive model. **a** H9C2 cells were treated with indoxyl sulfate (IS, 50 μg/mL), with or without probenecid (1 mM). **b** H9C2 cells were treated with p-cresyl sulfate (PCS, 100 μM), with or without probenecid (1 mM). Representative western blots and quantitative data are shown for fibronectin, E-cadherin, Bax, Bcl-2, p-p38/p38, and p-ERK/ERK. β-actin was used as a loading control. Quantitative data are shown in the corresponding graphs and presented as mean ± SEM (*n* = 3–6 per group). *p < 0.05, **p < 0.01, ***p < 0.001 vs. control; #*p* < 0.05, ##*p* < 0.01, ###*p* < 0.001 vs. IS + Probenecid 1 mM or PCS + Probenecid 1 mM.

Primary sinus node cells were analyzed separately as the principal pacemaker-relevant model. Primary sinus node cells exposed to IS (5 µg/mL) or PCS (100 µm) increased fibronectin and Bax expression while reducing Bcl-2 expression (shown in Fig. 3). IS and PCS also increased p-p38/p38 and p-ERK/ERK ratios, whereas TBX3 expression remained unchanged. These findings indicate the presence of profibrotic, apoptosis-associated, and stress kinase-associated molecular alterations in primary sinus node cells, with preservation of TBX3 expression as a pacemaker identity marker.

**Fig. 3.**
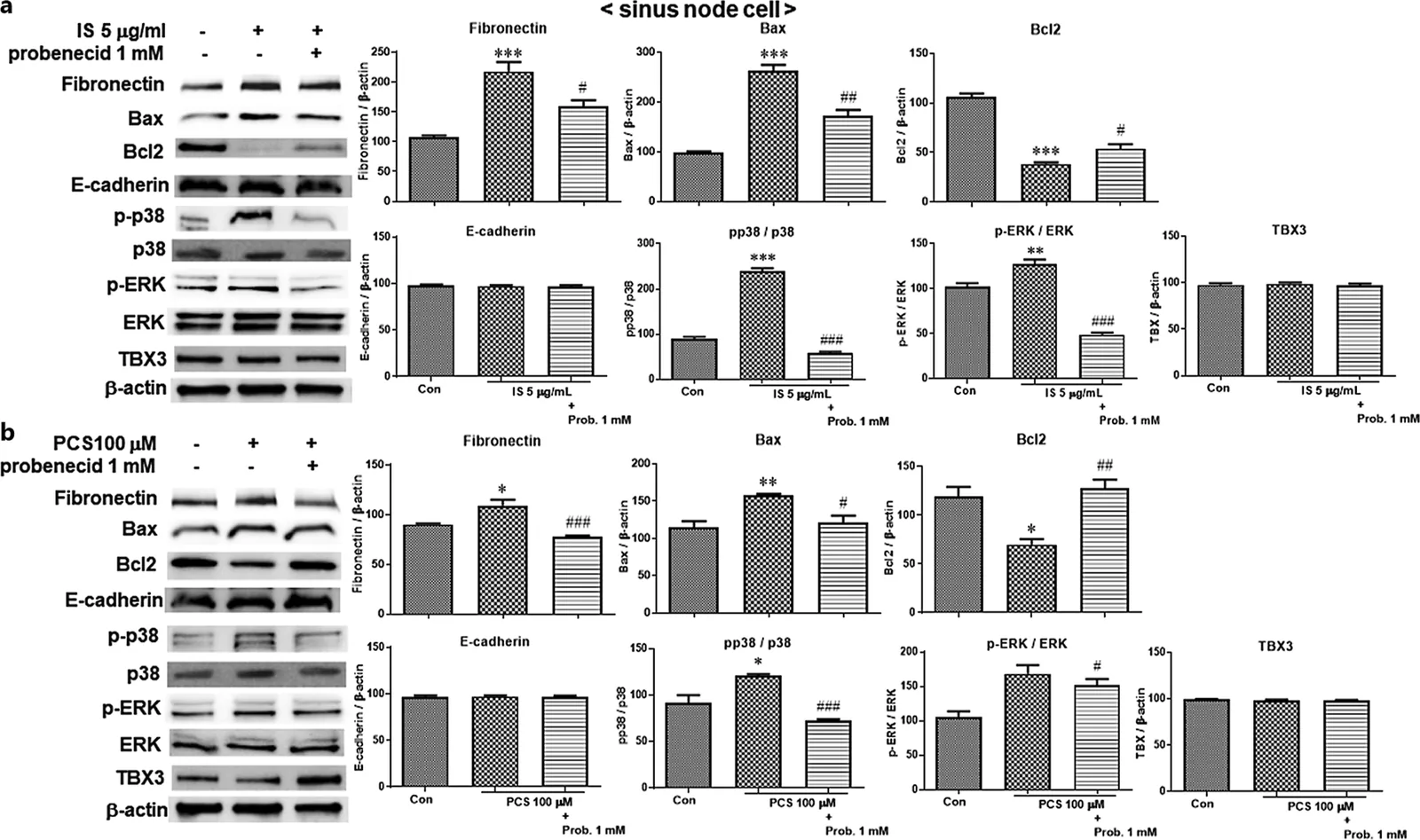
Effects of indoxyl sulfate, p-cresyl sulfate, and probenecid on profibrotic, apoptosis-associated, and stress-related molecular markers in primary sinus node cells. (**a**) Primary sinus node cells isolated from normal mouse hearts were treated with indoxyl sulfate (IS, 5 μg/mL), with or without probenecid (1 mM). (**b**) Primary sinus node cells isolated from normal mouse hearts were treated with p-cresyl sulfate (PCS, 100 μM), with or without probenecid (1 mM). Protein expression of fibronectin, Bax, Bcl-2, TBX3, p-p38/p38, and p-ERK/ERK was analyzed by western blotting. Quantitative data are shown in the corresponding graphs and presented as mean ± SEM (*n* = 3–6 per group). *p < 0.05, **p < 0.01, ***p < 0.001 vs. control; #*p* < 0.05, ##*p* < 0.01, ###*p* < 0.001 vs. IS + Probenecid 1 mM or PCS + Probenecid 1 mM.

In both experimental models, co-treatment with probenecid (1 mm) attenuated uremic toxin–associated molecular changes by reducing fibronectin and Bax expression, partially normalizing Bcl-2 expression, and suppressing p38 and ERK activation. However, the two models were interpreted differently: the H9C2 findings provided comparative evidence of generalized cardiomyocyte stress signaling, whereas conclusions regarding molecular alterations relevant to sinus node biology were derived primarily from the primary sinus node cell experiments. These marker-based findings should not be interpreted as direct evidence of functional impairment, apoptotic cell death, or loss of cell viability.

Uremic toxins induce generalized stress-associated gene expression in H9C2 cardiomyocytes and pacemaker-relevant molecular marker changes in primary sinus node cells. To determine whether the protein-level molecular alterations were accompanied by corresponding transcriptional responses, mRNA expression was analyzed in both H9C2 cardiomyocytes and primary sinus node cells following IS or PCS exposure. H9C2 cardiomyocytes were used as a comparative model of generalized cardiomyocyte stress responses, whereas primary sinus node cells served as the principal model for evaluating molecular alterations relevant to sinus node biology. In H9C2 cardiomyocytes, IS significantly increased mRNA expression of fibronectin, ANP, BNP, and β-MHC, indicating transcriptional activation of profibrotic and hypertrophy-associated gene programs (shown in Fig. 4a). PCS induced similar but less pronounced changes. Co-treatment with probenecid markedly attenuated toxin-induced upregulation of these genes, particularly fibronectin, ANP, and BNP. α-SMA mRNA expression showed only modest induction in response to IS or PCS, which was also reduced by probenecid. These findings indicate activation of generalized profibrotic and stress-associated transcriptional responses in H9C2 cardiomyocytes.

**Fig. 4.**
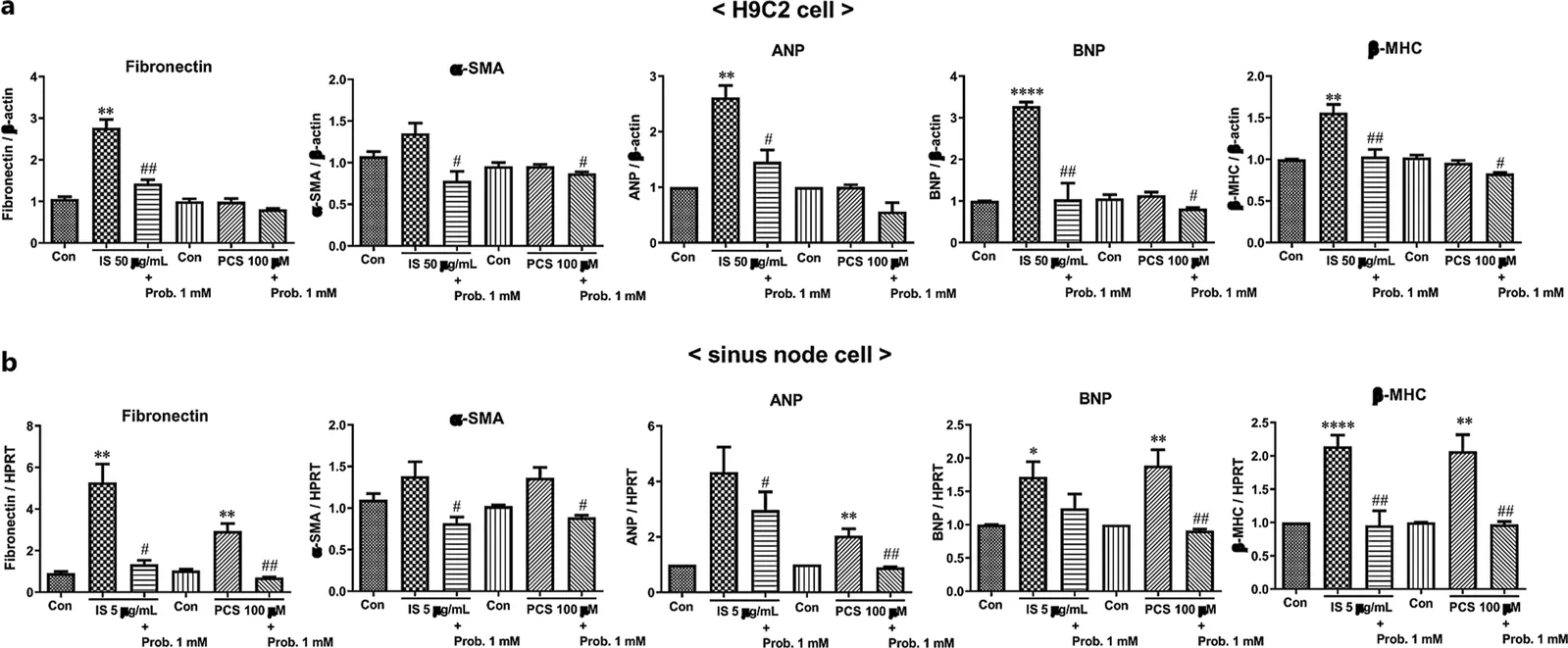
Transcriptional regulation of profibrotic, hypertrophy-associated, and molecular remodeling marker genes in H9C2 cardiomyocytes and primary sinus node cells following indoxyl sulfate or p-cresyl sulfate exposure with or without probenecid. (**a**) H9C2 cardiomyocytes were treated with indoxyl sulfate (IS, 50 μg/mL) or p-cresyl sulfate (PCS, 100 μM), in the presence or absence of probenecid (1 mM). (**b**) Primary sinus node cells were treated with IS (5 μg/mL) or PCS (100 μM), with or without probenecid (1 mM). mRNA expression levels of fibronectin, α-SMA, ANP, BNP, and β-MHC were analyzed by real-time PCR and normalized to HPRT. Data are presented as mean ± SEM (*n* = 3–5 per group). *p < 0.05, **p < 0.01, ***p < 0.0001 vs. control; #*p* < 0.05, ##*p* < 0.01 vs. IS + Probenecid 1 mM or PCS + Probenecid 1 mM.

Primary sinus node cells were analyzed separately as the principal pacemaker-relevant model. IS and PCS similarly upregulated fibronectin, ANP, BNP, and β-MHC transcripts (shown in Fig. 4b), indicating activation of remodeling-associated gene expression in this pacemaker-relevant model. Probenecid significantly attenuated these transcriptional changes, consistent with its effects observed at the protein level in primary sinus node cells.

Sinus node cells isolated from 2-week adenine-fed mice exhibit early uremic stress-associated molecular alterations that are attenuated by probenecid. To determine whether early uremic stress induced by adenine feeding is associated with molecular remodeling signatures in sinus node cells, sinus node cells were isolated from normal mice, mice fed a 0.2% adenine diet for 2 weeks [13]. Cells isolated from adenine-fed mice were subsequently treated ex vivo with probenecid (1 mm) under the same culture conditions. Compared with cells from normal mice, sinus node cells isolated from 2-week adenine-fed mice exhibited increases in fibronectin and Bax expression, accompanied by enhanced activation of p38 and ERK MAPK signaling (shown in Fig. 5). In contrast, Bcl-2 expression was reduced, while TBX3 levels remained unchanged, suggesting preservation of the pacemaker identity marker TBX3 despite activation of stress-associated molecular remodeling signatures. Treatment with probenecid (1 mm) attenuated fibronectin and Bax expression, partially normalized Bcl-2 levels, and suppressed p38 and ERK activation in sinus node cells isolated from adenine-fed mice.

**Fig. 5.**
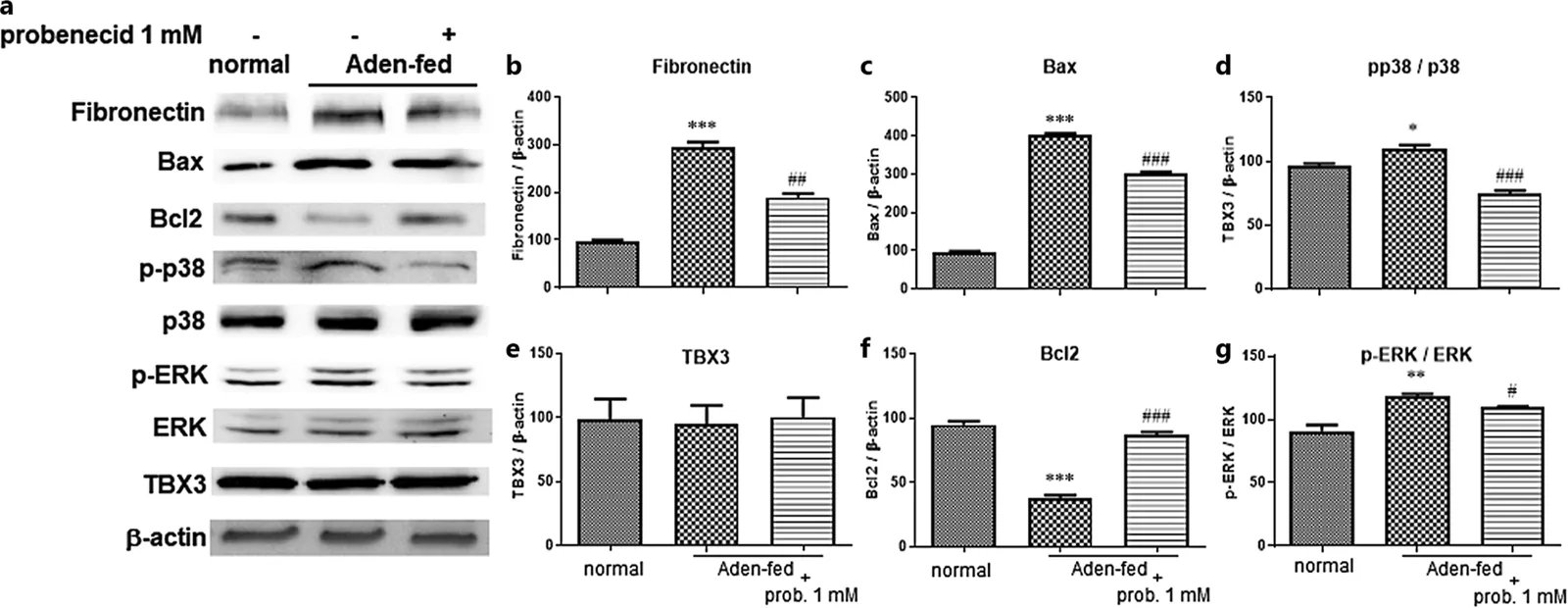
Effects of probenecid on profibrotic, apoptosis-associated, and molecular alterations in sinus node cells isolated from normal and 2-week adenine-fed mouse hearts. Primary sinus node cells were isolated from normal mice and mice fed a 0.2% adenine diet for 2 weeks, followed by culture under identical conditions. Sinus node cells from adenine-fed mice were subsequently treated with probenecid (1 mm) ex vivo. **a** Representative Western blot images of fibronectin, Bax, Bcl-2, p-p38, p38, p-ERK, ERK, TBX3, and β-actin. **b–g** Quantitative analyses of (**b**) fibronectin, (**c**) Bax, (**d**) p-p38/p38, (**e**) TBX3, (**f**) Bcl-2, and (**g**) p-ERK/ERK expression. Quantitative data are presented as mean ± SEM (*n* = 3 biologically independent experiments using primary sinus node cells isolated from individual mice per group). *p < 0.05, **p < 0.01 vs. normal; #*p* < 0.05, ##*p* < 0.01, ###*p* < 0.001 vs. Aden-fed + Prob. 1 mm.

Consistent with these protein-level findings, sinus node cells isolated from 2-week adenine-fed mice showed increased expression of fibronectin, α-SMA, ANP, BNP, and β-MHC transcripts (shown in Fig. 6). Ex vivo treatment with probenecid significantly attenuated these adenine-associated transcriptional changes, supporting its ability to attenuate uremic stress-associated molecular alterations in primary sinus node cells.

**Fig. 6.**
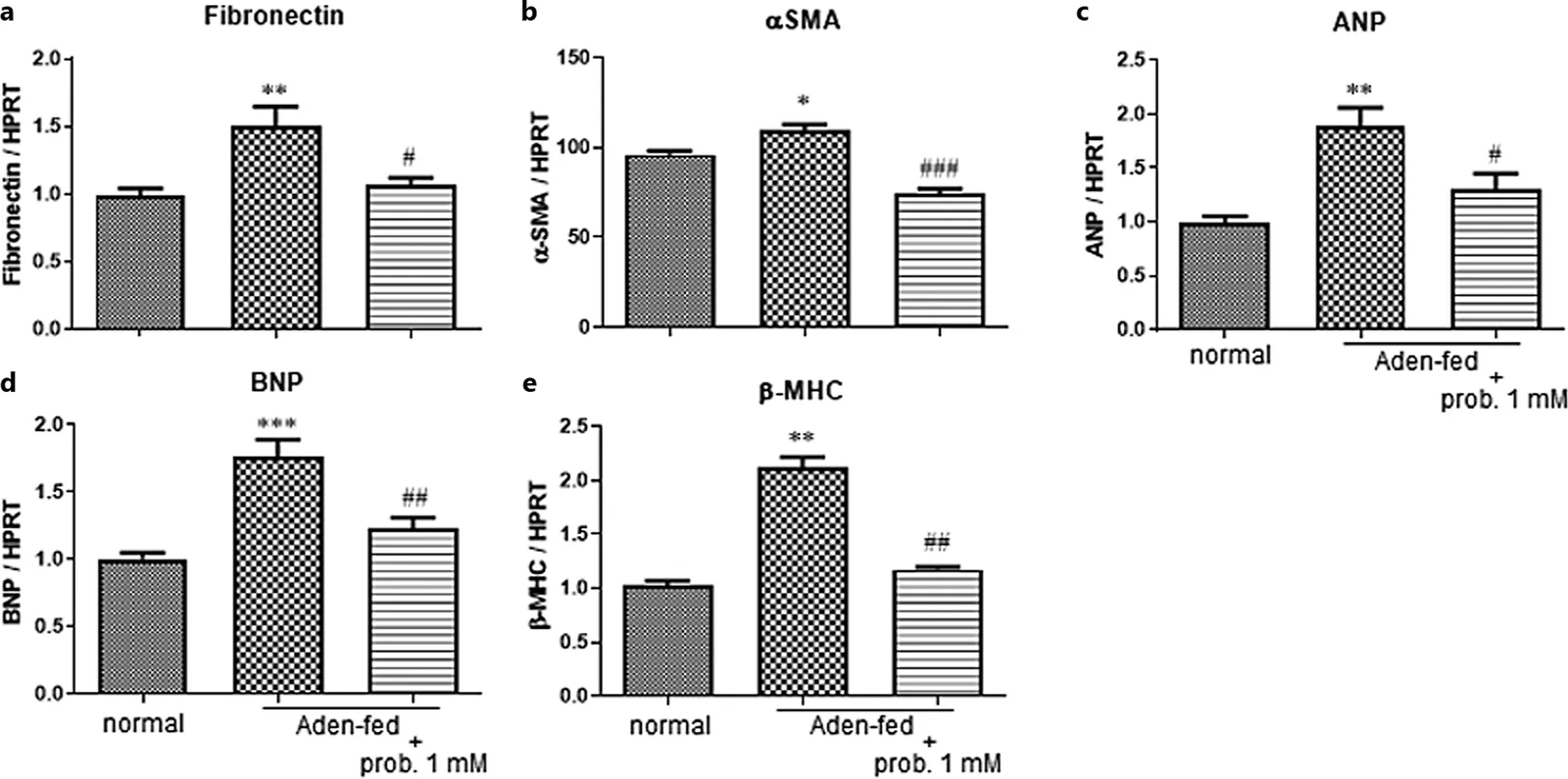
**a** fibronectin, **b** α-SMA, **c** ANP, **d** BNP, and **e** β-MHC are shown. Effects of probenecid on profibrotic, hypertrophy-associated, and molecular alteration marker expression in primary sinus node cells isolated from normal and 2-week adenine-fed mice. Primary sinus node cells were isolated from normal mice and mice fed a 0.2% adenine diet for 2 weeks, followed by culture under identical conditions. Primary sinus node cells isolated from adenine-fed mice were subsequently treated ex vivo with probenecid (1 mM). mRNA expression levels of (a) fibronectin, (b) α-SMA, (c) ANP, (d) BNP, and (e) β-MHC were analyzed by RT-qPCR and normalized to HPRT. Quantitative data are presented as mean ± SEM (n = 3 biologically independent experiments using primary sinus node cells isolated from individual mice per group). *p < 0.05, **p < 0.01 vs. normal; #*p* < 0.05, ##*p* < 0.01, ###*p* < 0.001 vs. Aden-fed + Prob. 1 mm.

## Discussion

This study provides one of the first comprehensive analyses of how the protein-bound uremic solutes IS and PCS, which accumulate in the CKD milieu, are associated with molecular alterations in sinus node cells that are relevant to cardiac pacemaker biology. While prior investigations of IS and PCS have focused predominantly on ventricular or vascular cells [14], the present work suggests that sinus node cells exhibit molecular remodeling responses under uremic toxin-associated stress. These findings provide a molecular framework for investigating how the uremic milieu may contribute to molecular alterations in sinus node cells in CKD.

IS and PCS elicited consistent profibrotic and apoptosis-associated molecular responses in both H9C2 cardiomyocytes and sinus node cells, characterized by increased fibronectin expression, elevated Bax, reduced Bcl-2, and activation of p38 and ERK signaling. These findings suggest that uremic toxins activate a shared cardiac stress-responsive signaling program. However, because H9C2 cells do not represent bona fide pacemaker cells, the H9C2 data were interpreted only as comparative evidence of general cardiac stress signaling. In contrast, the findings in primary sinus node cells provide the primary basis for interpreting pacemaker-relevant molecular alterations. Importantly, these changes were assessed as molecular markers of profibrotic and pro-apoptotic signaling, and they do not by themselves establish functional impairment, apoptotic cell death, or histological fibrosis. Notably, sinus node cells exhibited marked upregulation of remodeling-associated molecular markers while maintaining stable TBX3 expression, suggesting that these molecular alterations occurred without overt loss of this pacemaker identity marker. This observation supports the possibility that molecular signaling alterations may precede overt changes in pacemaker cell identity.

The use of different IS concentrations in H9C2 cardiomyocytes (50 μg/mL) and primary sinus node cells (5 μg/mL) was intended to account for cell type–specific tolerance to IS exposure. H9C2 cardiomyocytes were treated with 50 μg/mL IS based on their higher tolerance and previous H9C2-based studies using comparable or higher IS concentrations to evaluate uremic toxin-induced cardiac stress signaling [15, 16]. In contrast, primary sinus node cells showed greater vulnerability to IS exposure during preliminary culture observations; therefore, 5 μg/mL IS was selected as a working concentration that allowed assessment of toxin-associated molecular responses while minimizing nonspecific cell loss. Because formal dose-response cytotoxicity assays were not performed, the different IS concentrations used in H9C2 cardiomyocytes and primary sinus node cells should not be interpreted as directly comparable in vivo equivalents, but rather as cell type–specific working concentrations selected to permit interpretable molecular analyses.

Importantly, sinus node cells isolated from 2-week adenine-fed mice exhibited molecular alterations consistent with our in vitro findings. These alterations included increased fibronectin and Bax expression, enhanced MAPK activation, and corresponding increases in remodeling-associated transcripts. However, this short-term adenine-fed model should be interpreted as an early uremic stress model rather than a model of advanced chronic CKD-associated sinus node remodeling. Accordingly, these ex vivo findings support the presence of uremia-related molecular changes in sinus node cells, but they do not establish whether these molecular alterations lead to impaired sinus node function in vivo. Additional studies incorporating ECG recordings, heart rate measurements, and electrophysiological assessment of pacemaker activity will be required to determine whether these molecular alterations translate into functional abnormalities of the sinus node in vivo.

A key finding of this study is that probenecid attenuated uremic toxin–associated molecular stress and remodeling signatures. Probenecid has been reported to modulate cardiac Ca^2+^ handling through TRPV2-related mechanisms [10, 17, 18]. In addition, probenecid has been widely used as an inhibitor of organic anion transport, and OAT1/OAT3-mediated pathways are known to contribute to the handling of uremic toxins and solutes [19, 20]. Therefore, one possible explanation is that probenecid may reduce uremic toxin-associated intracellular stress signaling by affecting organic anion transporter-mediated toxin handling. Uremic toxins have also been linked to oxidative stress, inflammatory signaling, mitochondrial dysfunction, and ROS/MAPK-mediated cardiac remodeling, which may converge on p38 and ERK activation [6, 7]. However, intracellular toxin levels, transporter activity, ROS generation, and TRPV2-dependent signaling were not directly measured in the present study. Therefore, these mechanisms should be interpreted as plausible explanations rather than definitive causal pathways. Future studies using pharmacological inhibition of MAPK signaling, upstream ROS pathway analysis, transporter inhibition or knockdown, and measurement of intracellular uremic toxin accumulation will be required to define the causal mechanisms underlying these molecular responses.

Clinically, unexplained bradycardia, low chronotropic response, and nodal conduction delay are common yet therapeutically unresolved problems in CKD [21, 22]. By identifying uremic toxin-associated MAPK activation as part of the molecular alterations observed in sinus node cells and showing that probenecid attenuates these marker changes, this study provides a rationale for further investigation of strategies aimed at preserving sinus node cell homeostasis and, potentially, SAN function in renal disease.

Several limitations should be acknowledged. First, H9C2 cardiomyocytes are not pacemaker cells and do not recapitulate sinus node automaticity. Therefore, H9C2 data should be interpreted as comparative cardiac stress signaling data, whereas conclusions regarding pacemaker-relevant molecular alterations rely primarily on primary sinus node cell findings, including cells isolated from 2-week adenine-fed mice. Second, the present study primarily assessed molecular markers associated with profibrotic signaling, apoptosis-associated signaling, and stress kinase activation, including fibronectin, Bax, Bcl-2, p38, and ERK. Therefore, these findings should be interpreted as molecular stress and remodeling signatures rather than direct evidence of functional impairment, apoptotic cell death, or histological fibrosis. Third, the 2-week adenine-fed mouse model used in this study should be interpreted as an early uremic stress model rather than a model of advanced CKD-associated molecular alterations in the sinus node. Because ECG recordings, heart rate measurements, and electrophysiological assessment of pacemaker activity were not performed, the present data do not establish whether the observed molecular alterations translate into functional abnormalities of the sinus node in vivo. Fourth, electrophysiological properties of isolated sinus node cells, including ion currents, pacemaker activity, and Ca^2+^ cycling, were not directly assessed. Fifth, the protein-level measurements were obtained at a single experimental time point, which limits our ability to distinguish transient adaptive responses from sustained pathological remodeling. Future studies incorporating viability assays, apoptosis assays, histological assessment of fibrosis, time-course analyses, patch-clamp-based functional analyses, prolonged CKD models, ECG recordings, heart rate measurements, and in vivo rhythm assessments will be required to determine the temporal progression, functional significance, and therapeutic potential of probenecid.

In summary, this study shows that sinus node cells exhibit profibrotic, apoptosis-associated, and stress-responsive molecular alterations in response to uremic toxin-induced exposure, and that these molecular signatures are attenuated by probenecid. These findings provide a molecular basis for further investigating CKD-associated molecular alterations in sinus node cells. Importantly, our findings are supported by clinical observations showing that patients with CKD have an increased incidence of bradyarrhythmias, conduction abnormalities, and permanent pacemaker implantation, suggesting that uremic toxin-associated molecular stress may contribute to the vulnerability of the cardiac conduction system. Although the present study does not establish a direct causal relationship between these molecular alterations and functional abnormalities of the sinus node, it provides mechanistic evidence supporting future investigations into the role of uremic toxin-responsive signaling pathways in preserving sinus node cell homeostasis in CKD.

## Statement of Ethics

This study protocol was reviewed and approved by the Institutional Animal Care and Use Committee of Ewha Womans University (Approval No.: 21-020). All animal procedures were conducted in accordance with the National Institutes of Health (NIH) Guide for the Care and Use of Laboratory Animals.

## Conflict of Interest Statement

The authors declare that they have no conflicts of interest.

## Funding Sources

This research was supported by the Basic Science Research Program through the National Research Foundation of Korea (NRF), funded by the Ministry of Education (NRF-2021R1I1A1A01041710). This work was supported by the National Research Foundation of Korea (NRF) grants (2020R1A5A2019210 and 2023R1A2C2003185) funded by the Korea Ministry of Science and ICT. The funders had no role in the design, data collection, data analysis, and reporting of this study.

## Author Contributions

K.H.K. conceived and designed the study, supervised all experimental procedures, and drafted the initial version of the manuscript. D.-A.K. performed conceptualization and data analysis under the supervision of K.H.K. C.H.J. contributed to investigation, methodology, and project administration. J.-H.R. contributed to formal analysis and funding acquisition. M.-R.L. contributed to software, supervision, validation, and final approval of the manuscript. All authors read and approved the final manuscript.

## Data Availability

The data that support the findings of this study are not publicly available due to data management considerations but are available from the corresponding author, K.H.K., upon reasonable request.
